# Insights into myopic choroidal neovascularization based on quantitative proteomics analysis of the aqueous humor

**DOI:** 10.1186/s12864-023-09761-z

**Published:** 2023-12-12

**Authors:** Huimin Yu, Zheng Zhong, Yin Zhao, Huan Luo, Jinfu Sun, Ruohong Wang, Xian Zhang, Xufang Sun

**Affiliations:** 1 Department of Ophthalmology, Tongji Hospital, Tongji Medical College, Huazhong University of Science and Technology, 1095 Jie-fang Road, Wuhan, Hubei Province China; 2 School of Optometry and Ophthalmology and Eye Hospital, Wenzhou Medical University, Wenzhou, Zhejiang Province, China

**Keywords:** Proteomic, Pathologic myopia, Choroidal neovascularization, Aqueous humor, GFAP, Biomarker

## Abstract

**Background:**

Previous studies on the biomarkers of pathologic myopia choroidal neovascularization (pmCNV) development merely detected limited types of proteins and provide a meagre illustration of the underlying pathways. Hence, a landscape of protein changes in the aqueous humor (AH) of pmCNV patients is lacking. Here, to explore the potential mechanisms and biomarkers of pmCNV, we analyzed the clinical data and protein profile among atrophic (A) lesions, tractional lesions (T) and neovascular (N) lesions in myopic patients based on the ATN grading system for myopic maculopathy (MM).

**Results:**

After investigating demographic data of our patients, a correlation was found between A and N lesions (R = 0.5753, P < 0.0001). Accordingly, groups were divided into patients without MM, patients with myopic atrophic maculopathy (MAM), and patients with pmCNV (N2a lesion). In proteomics analysis, the increased protein level of GFAP and complement-associated molecules in AH samples of the 3 groups also indicated that MAM and pmCNV shared similar characteristics. The GO enrichment and KEGG pathway analysis were performed, which mapped that differential expressed proteins mainly engaged in JAK-STAT pathway between the pmCNV group and two controls. Furthermore, we identified several potential biomarkers for pmCNV, including FCN3, GFAP, EGFR, SFRP3, PPP2R1A, SLIT2, and CD248.

**Conclusions:**

Atrophic lesions under pathologic myopic conditions demonstrated similarities to neovascularization development. Potential biomarkers including GFAP were associated with the pathogenesis of pmCNV. In summary, our study provides new insights for further research on pmCNV development.

**Supplementary Information:**

The online version contains supplementary material available at 10.1186/s12864-023-09761-z.

## Background

Pathological myopia (PM)-associated complications have gradually become the major cause of the impairment or loss of visual acuity in highly myopic populations [1]. The prevalence of pathologic myopia is approximately 0.9-3.1% and the prevalence of visual impairment is 0.2-1.4% Asian population [2]. The five-year incidence of myopic maculopathy was 0.05% in rural Chinese adults, while another study reports a five-year accumulative incidence of 0.3% in a European population [3, 4]. Choroidal neovascularization (CNV), considered one of the most severe complications of PM, has a progressive and devastating effect on vision [5]. Although anti-vascular endothelial growth factor (VEGF) therapy has replaced photodynamic therapy, pmCNV remains an urgent challenge for low responders and a burden for patients who need multiple treatments or among patients with severe myopic maculopathy [5, 6]. Remarkably, the efficacy of anti-VEGF drugs, such as ranibizumab, may depend on genetic polymorphisms, in which FLT1 (rs9582036, rs7993418) variants may serve as a genetic marker [7]. In fact, other risk factor, including the presence of perforating scleral vessels or macular Bruch membrane breakdown, may hinder the response to anti-VEGF therapy as well [8, 9]. Accordingly, other underlying mechanisms and biomarkers for pmCNV remain to be revealed, which could offer possibilities for novel treatment strategies.

Aqueous humor (AH) connects between the anterior and posterior chambers, and is composed of metabolic products and proteins derived from different segments of the eye. Proteomic investigation of AH is an essential step toward deciphering the functional basis and deepening our understanding of pathological mechanisms in diseases such as aged-related macular degeneration (AMD) [10], diabetic retinopathy [11], and myopia [12]. Several previous studies reported proteomics analyses concerning the pathogenesis of myopia [13–15], which provided clues about the biomarkers during the development of myopia. However, omics data from pmCNV patients have rarely been reported.

One of the hypotheses of pmCNV formation is that the activation of hypoxia associated pathways might induce the process of neovascularization [5, 16]. However, the mechanism of pmCNV has not been fully elucidated. Previous studies on biomarkers of pmCNV development are limited to the minor types of proteins detected and provided a meagre illustration of the underlying pathways [17–23].

ATN classification system provided a more precise approach for myopic maculopathy. The current atrophy classification integrated existing standards, and the tractional component described the features of foveoschisis and macular hole. The neovascularization component focused on characterization of lacquer cracks/CNV/Fuch’s spots. Taking advantage of the ATN grading system for myopic maculopathy [24], the well-defined stages might provide more specific information in the process of pmCNV. In our study, the correlation between atrophic lesions and neovascular lesions in the ATN grading system was analyzed; a label-free quantitative proteomics method and subsequent bioinformatic analysis were employed to reveal the development of pmCNV in the AH of our patients. We aimed to explore the similarities or distinctions of the protein expression profile during the different stages of PM and demonstrate more possible biomarkers for pmCNV.

## Results

### Demographic data of the myopic cohort

**Fig. 1:**
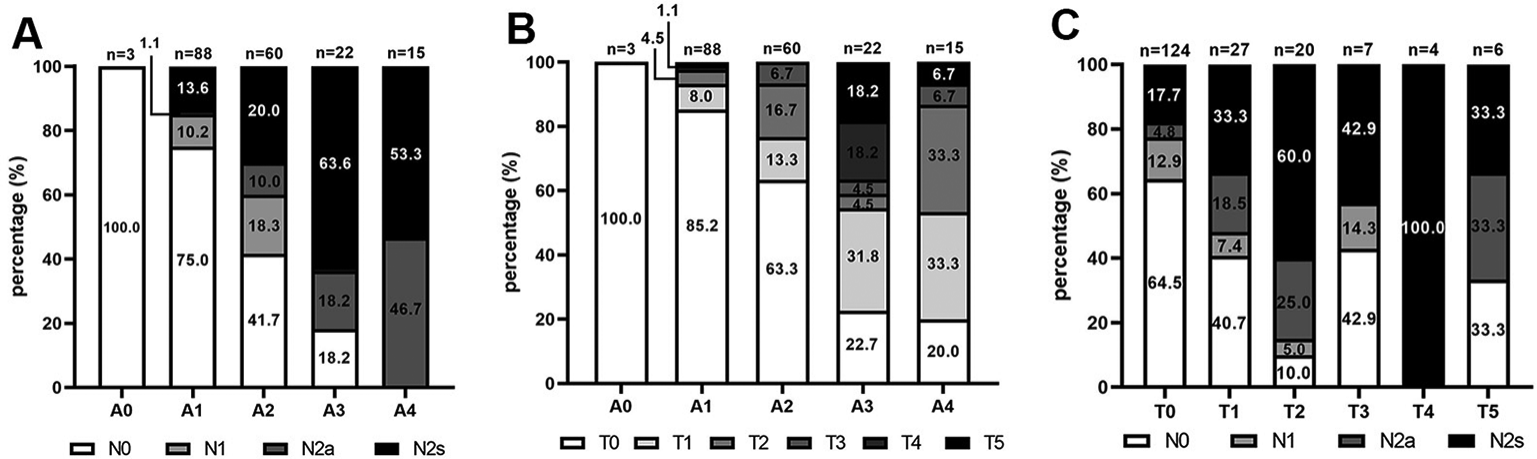
Distribution of different groups of myopic maculopathy according to ATN grading system in a highly myopic cohort. (**A**) Distribution of Neovascular (N) type stratified by Atrophic (**A**) type (**B**) Distribution of Tractional (T) type stratified by Atrophic (**A**) type (**C**) Distribution of Neovascular (N) type stratified by Tractional (T) type

We first analyzed the correlation between the two types of myopic maculopathy in 188 previously enrolled highly myopic eyes (117 participants) [25] (Table 1). Myopic maculopathy (MM) included three components: atrophic lesions (A), tractional lesions (T), and neovascular lesions (N). Spearman correlation analysis revealed that A and N were positively correlated (R = 0.5603, P < 0.0001). Figure 1 also shows that with the enlargement of the A lesion, the proportion of eyes with a higher N lesion increased. However, the correlations between A and T (R = 0.4905, P < 0.0001) and between T and N (R = 0.4087, P < 0.0001) were weaker.

**Table 1:** Correlation between different groups of myopic maculopathy

Group		Spearman R		
		R Value	95% CI	P Value
A vs. N		0.5603	0.4502 to 0.6536	< 0.0001
A vs. T		0.4905	0.3699 to 035948	< 0.0001
T vs. N		0.4087	0.2781 to 0.5244	< 0.0001

CI, confidence interval. R and P values were derived from Spearman correlation test

Twenty-four aqueous humor samples from 24 treatment naïve highly myopic eyes were collected. These samples were divided into 3 groups, including the non-MM (G3) group, myopic atrophic maculopathy (MAM) (G2) group, and pmCNV (namely neovascular stage2a) (G1) group, for further proteomic experiments. Clinical characteristics between pmCNV eyes and non-CNV eyes are compared in Table 2. The G1 group (pmCNV) was older than the G2 (MAM) and G3 (non-MM) groups (P-g1g2 = 0.007 and P-g1g3 = 0.009, respectively). There was no difference in AL (P-g1g2 = 0.261 and P-g1g3 = 0.092, respectively), sex (P-g1g2 = 1.000 and P-g1g3 = 0.627, respectively), bilateral (P-g1g2 = 0.638 and P-g1g3 = 0.638, respectively), or T lesion (P-g1g2 = 0.057 and P-g1g3 = 0.070, respectively).

**Table 2:** Comparison of clinical characteristics between pmCNV eyes and non-CNV eyes

	G1 group (pmCNV)	G2 group (MAM)	G3 group (non-MM)	P-g1g2	P-g1g3	
No. of eyes	12	6	6			
Age	61.17 ± 9.54	48.50 ± 3.62	47.17 ± 6.11	0.007		0.009
AL	29.61 ± 1.84	31.18 ± 3.12	28.19 ± 1.95	0.261		0.092
Gender/Female	8 (66.7%)	4 (66.7%)	3 (50.0%)	1.000		0.627
Bilateral/OD	6 (50.0%)	2 (33.3%)	4 (66.7%)	0.638		0.638
A lesion				0.308		0.006
0	0 (0.0%)	0 (0.0%)	2 (33.3%)			
1	1 (8.3%)	0 (0.0%)	4 (66.7%)			
2	4 (33.3%)	4 (66.7%)	0 (0.0%)			
3	3 (25.0%)	2 (33.3%)	0 (0.0%)			
4	4 (33.3%)	0 (0.0%)	0 (0.0%)			
T lesion				0.057		0.070
0	5 (41.7%)	6 (100.0%)	6 (100.0%)			
1	5 (41.7%)	0 (0.0%)	0 (0.0%)			
2	2 (16.7%)	0 (0.0%)	0 (0.0%)			
N lesion				< 0.001		< 0.001
0	0 (0.0%)	6 (100.0%)	6 (100.0%)			
2a	12 (100.0%)	0 (0.0%)	0 (0.0%)			

P-g1g2 was derived from a comparison between the G1 group (pmCNV) and the G2 group (MAM).

P-g1g3 was derived from a comparison between the G1 group (pmCNV) and the G3 group (non-MM).

### Differentially expressed proteins among the pmCNV and control myopic groups

**Fig. 2:**
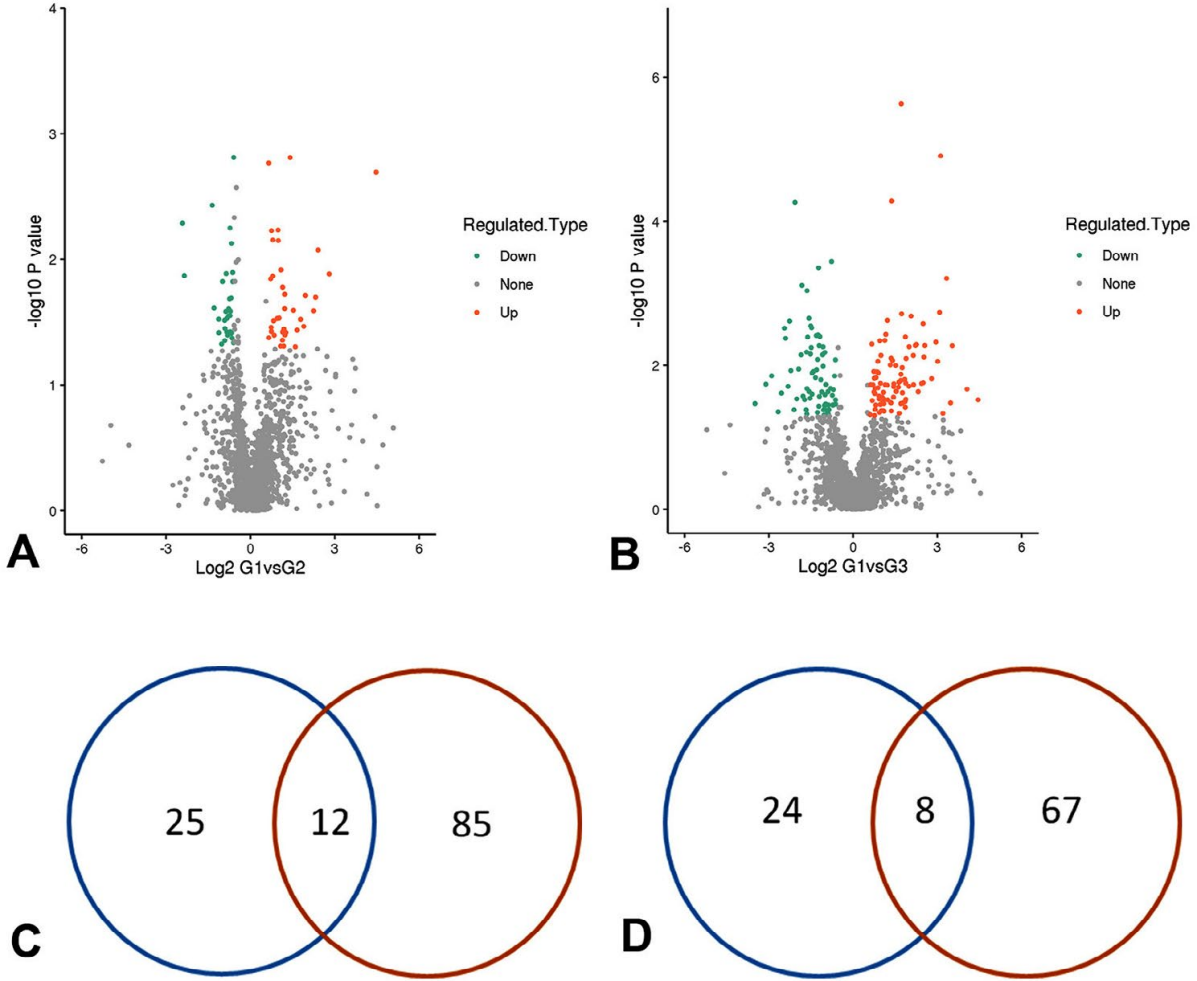
Comparison between pmCNV and the controls. (A-B) Green points refer to downregulated proteins; orange points refer to upregulated proteins. Comparisons were performed between the G1 (pmCNV) group and the G2 (MAM) group (**A**) and between the G1 group and the G3 (non-MM) group (**B**). (**C-D**) Venn diagrams of the number of DEPs. The blue circle refers to the comparison between the G1 group and G2 group, and the red circle refers to the comparison between the G1 group and G3 group. In the overlapping parts, 12 DEPs were upregulated (**C**), and 8 DEPs were downregulated (**D**)

**Fig. 3:**
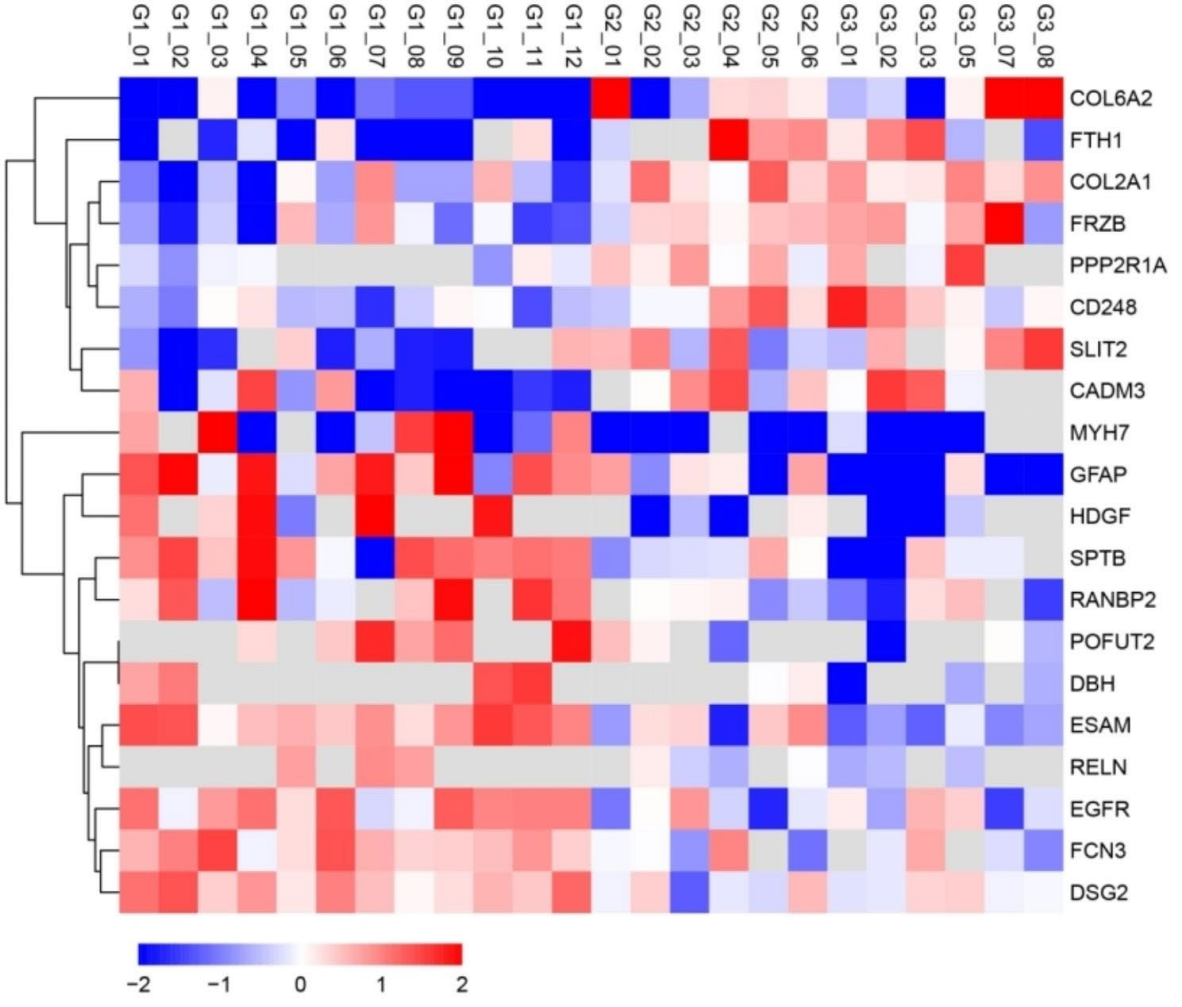
Heatmap of 20 overlapping DEPs. G1 refers to aqueous humor from pmCNV patients. G2 refers to aqueous humor from MAM patients. G3 refers to aqueous humor from non-MM patients

The total proteins identified in our study were 1737, and the 1654 proteins were quantifiable. To explore aqueous humor biomarkers, comparisons were performed between pmCNV and the two control groups (Fig. 2). In the comparison between G1 (pmCNV) and G2 (MAM), 37 differentially expressed proteins (DEPs) were upregulated, and 32 DEPs were downregulated. In the comparison between G1 and G3 (non-MM), 97 DEPs were upregulated, and 75 DEPs were downregulated. The overlapping DEPs in these two comparisons were further analyzed. There were 12 overlapping upregulated DEPs, including desmoglein-2 (DSG2), ficolin-3 (FCN3), epidermal growth factor receptor (EGFR), reelin (RELN), E3 SUMO-protein ligase (RANBP2), spectrin beta chain (SPTB), endothelial cell-selective adhesion molecule (ESAM), peptide-O-fucosyltransferase (POFUT2), dopamine beta-hydroxylase (DBH), hepatoma-derived growth factor (HDGF), glial fibrillary acidic protein (GFAP), and myosin (MYH7). There were 8 overlapping downregulated DEPs, including ferritin heavy chain (FTH1), collagen alpha-2(VI) (COL6A2), SLIT homolog 2 protein (SLIT2), cell adhesion molecule 3 (CADM3), collagen alpha-2(VI) (COL2A1), endosialin (CD248), secreted frizzled-related protein 3 (SFRP3), and serine/threonine-protein phosphatase 2 A regulatory subunit A alpha (PPP2R1A). A heatmap of the 20 overlapping DEPs is shown in Fig. 3.

Additionally, DEPs between the G2 (MAM) group and the G3 (non-MM) group were analyzed (Supplementary Figure S1). There were 32 upregulated proteins and 39 downregulated proteins, and the relative intensities of these DEPs are shown in Supplementary Table S1 and Supplementary Table S2.

### GO and KEGG enrichment analysis

**Fig. 4:**
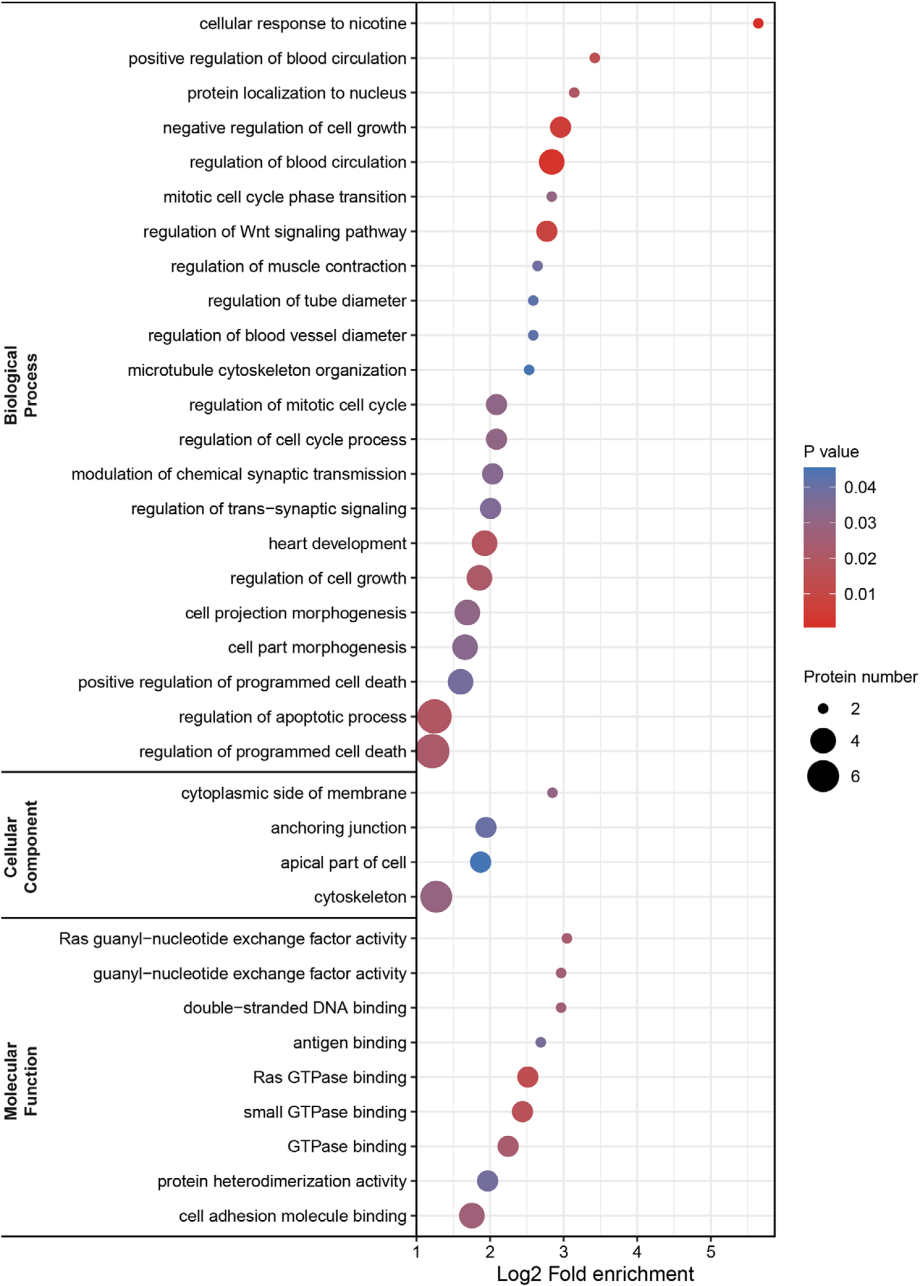
Bubble map of GO enrichment analysis. GO annotation was composed of three parts: biological process, cellular component, and molecular function. Bubble size was associated with the number of proteins, and bubble color represents different P values

DEPs were classified by Gene Ontology (GO) annotation into three categories: biological process, cellular component, and molecular function (Fig. 4). Our results revealed that several biological processes were involved in pmCNV, including regulation of blood circulation, the Wnt signaling pathway, cell growth and cell death, as well as cellular response to nicotine. In the cellular component category, DEPs were related to the cytoskeleton and anchoring junction. In molecular function, DEPs were associated with GTPase binding, cell adhesion molecule binding, and so forth. The underlying signaling pathways of pmCNV were also analyzed by Kyoto Encyclopedia of Genes and Genomes (KEGG) annotation, which demonstrated activation of the JAK-STAT signaling pathway, protein digestion and absorption, the PI3K-Akt signaling pathway, and focal adhesion (Table 3).

**Table 3:** KEGG enrichment analysis. The P value was derived from Fisher’s exact test

KEGG pathway	Fold Enrichment	-log10 (P value)	Proteins
hsa04630 JAK-STAT signaling pathway	17.69	2.32	EGFR, GFAP, PPP2R1A
hsa05165 Human papillomavirus infection	5.16	2.29	COL6A2, COL2A1, EGFR, PPP2R1A
hsa04151 PI3K-Akt signaling pathway	3.81	1.81	COL6A2, COL2A1, EGFR, PPP2R1A
hsa05160 Hepatitis C	8.85	1.71	EGFR, PPP2R1A
hsa04261 Adrenergic signaling in cardiomyocytes	8.26	1.65	PPP2R1A, MYH7
hsa04510 Focal adhesion	3.72	1.39	COL6A2, COL2A1, EGFR
hsa04974 Protein digestion and absorption	5.63	1.34	COL6A2, COL2A1

### Potential biomarkers in pmCNV

**Fig. 5:**
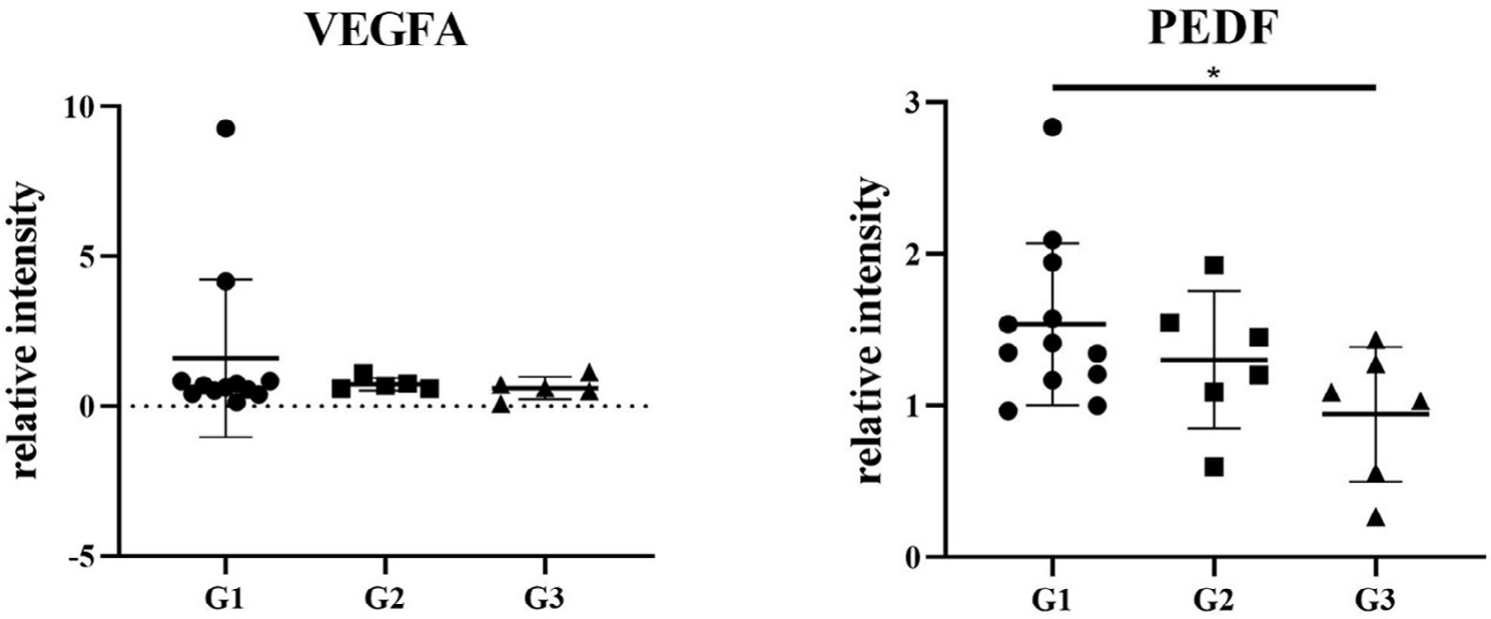
The relative intensity of VEGFA and PEDF among the 3 groups *P < 0.05

Supplementary Table S3 summarizes pmCNV biomarkers in previous studies of treatment naïve patients [17–23]. The source of the sample differed from aqueous humor to serum and vitreous humor. The detection methods varied, including ELISA, human cytokine panels, and proteomics. VEGF and pigment epithelial derived factor (PEDF) were reported in the majority of previous studies, but the trend of those proteins in treatment naïve pmCNV samples compared to the control group greatly differed (Supplementary Table S3) [17–21]. Figure 5 demonstrates the relative intensity of VEGFA and PEDF in our study. In comparison with previous reports, we observed that the relative intensity of VEGFA was not affected among the 3 myopic groups (P > 0.05). The relative intensity of PEDF was higher in the G1 group than in the G3 group (P = 0.019), but no difference was found between the G1 and G2 groups (P = 0.314) or between the G2 and G3 groups (P = 0.225).

**Fig. 6:**
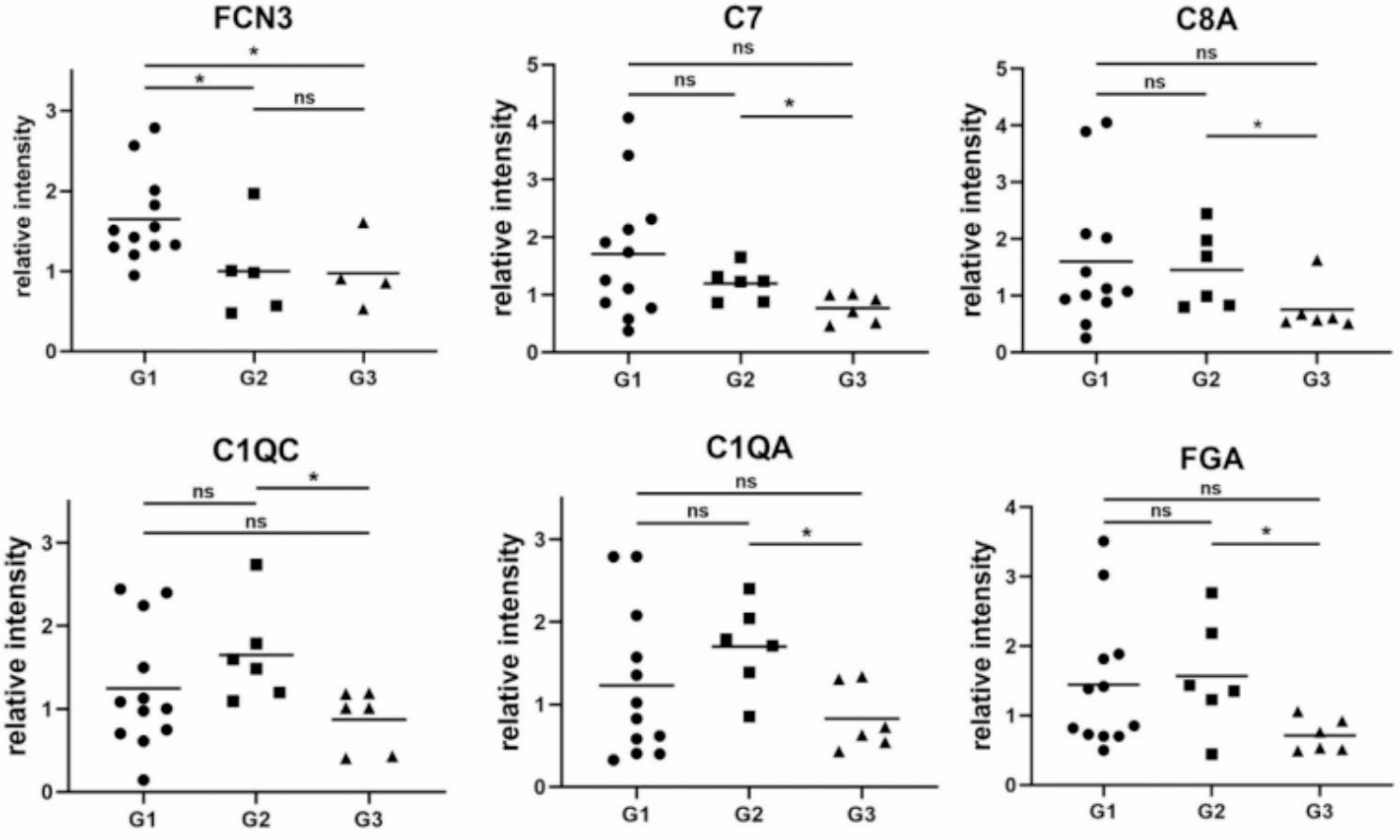
The relative intensity of proteins in the complement component *P < 0.05; ns, not significant

Proteins related to complement components were also shown to be related to MAM and pmCNV progression. Ficolin-3 (FCN3) might also be a potential biomarker for pmCNV since it was highly upregulated in the aqueous humor of pmCNV patients compared with controls (G1 higher than G2, P = 0.0143; G1 higher than G3, P = 0.0151) (Fig. 6). Notably, other proteins related to the complement component were also upregulated in the MAM group compared with the non-MM group, including C7 (P = 0.0244), C8A (P = 0.0325), C1QC (P = 0.0192), C1QA (P = 0.0103) and fibrinogen alpha chain (FGA) (P = 0. 0392) (Fig. 6, Supplementary Table S1).

**Fig. 7:**
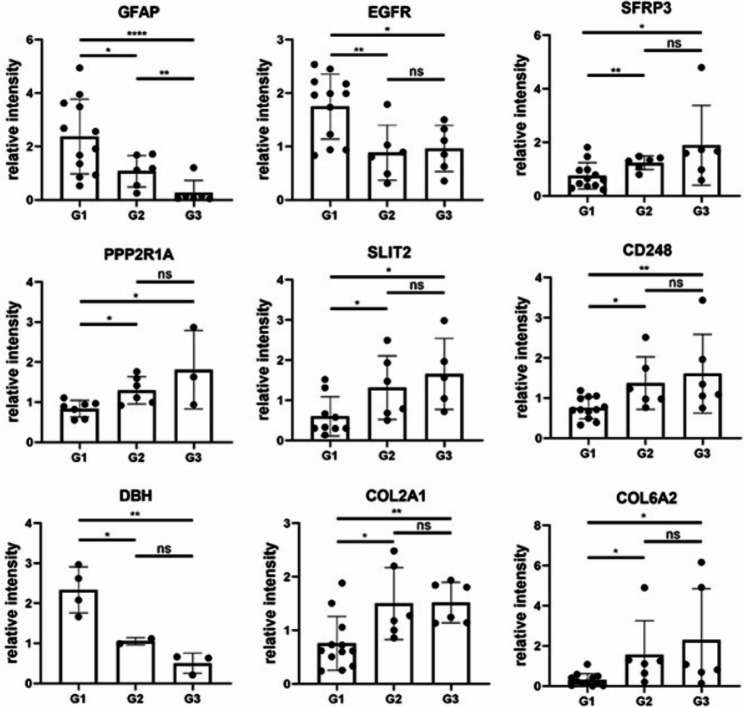
The relative intensity of 10 potential biomarkers for pmCNV. *P < 0.05; **P < 0.01; ***P < 0.001; ***P < 0.0001; ns, not significant

Based on a comprehensive research of the literature, several proteins might serve as biomarkers (Fig. 7). The relative intensity of GFAP gradually increased as the lesion of MM developed (G1 higher than G2, P = 0.0439; G2 higher than G3, P = 0.0066). The relative intensities of EGFR, SFRP3, PPP2R1A, SLIT2, CD248, DBH, COL2A1, and COL6A2 were different between the G1 and G2 groups and between the G1 and G3 groups (P < 0.05) but were not different between the G2 and G3 groups (P > 0.05).

### Correlation among potential AH biomarkers and clinical parameters

**Fig. 8:**
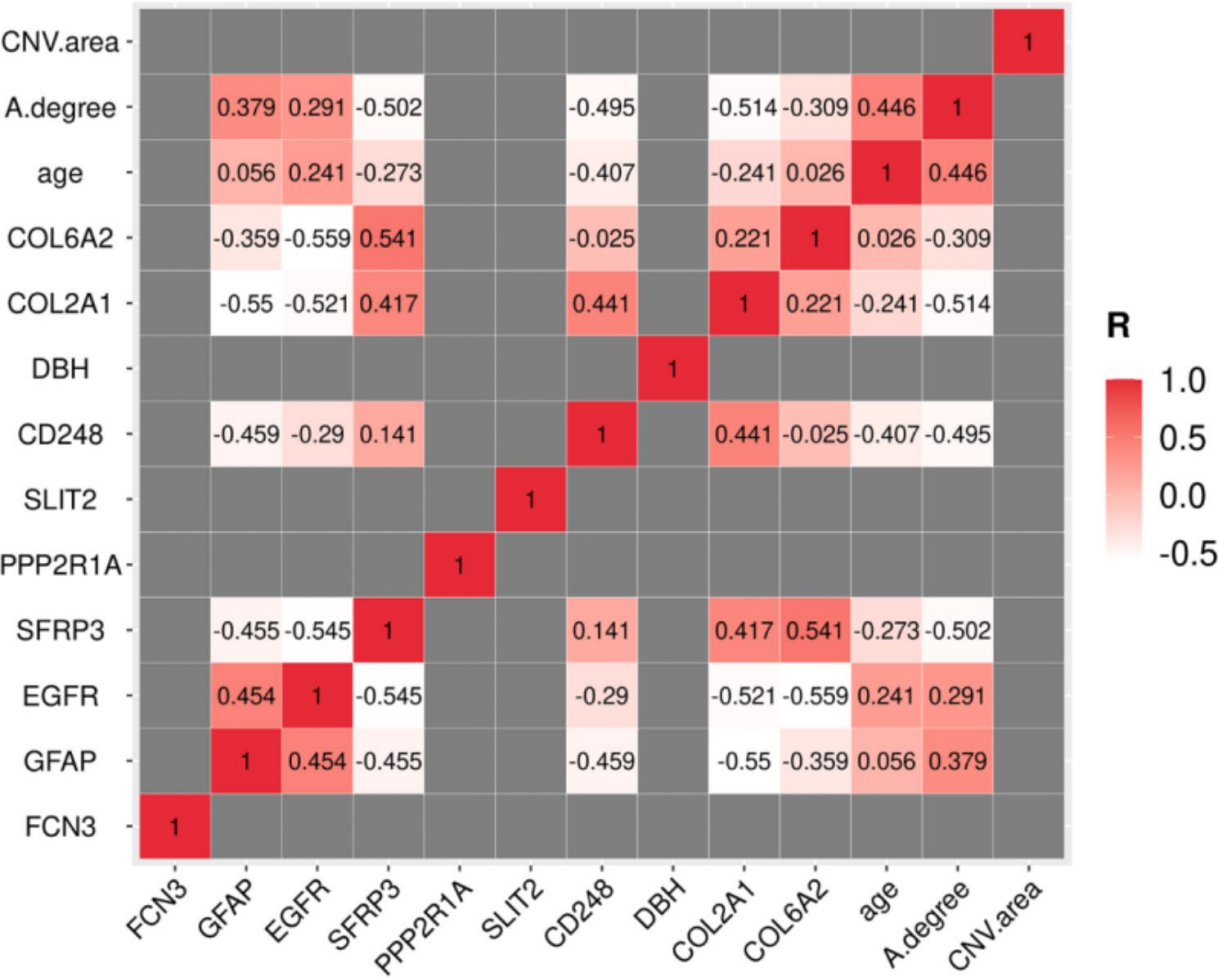
The correlation heatmap of potential biomarkers and clinical parameters. Correlations among different proteins and correlations between those proteins and clinical parameters (age, A degree, CNV area) were analyzed. P and R values were derived from correlation tests. If P > 0.05, the box in the heatmap was drawn gray; if P < 0.05, the box contained the R value and was drawn red if R > 0 and white if R < 0

Clinical data and profile of potential biomarkers were catalogued in detail. (Supplementary Table S4 and Supplementary Table S5). Since our demographic analysis revealed that pmCNV group were older than in the control group (Table 2), a correlation between age and potential biomarkers was performed to elucidate the impact of age on these biomarkers (Fig. 8). We found correlations between age and FCN3 (P > 0.05), GFAP (R = 0.056 < 0.2), PPP2R1A (P > 0.05), SLIT2 (P > 0.05), DBH (P > 0.05), COL6A2 (R = 0.026 < 0.2), and CNV area (P > 0.05) that were not significant. Age and EGFR were positively correlated to a weak degree (R = 0.241), and a weak negative correlation between age and SFRP3 (R = -0.273) and between age and COL2A1 (R = -0.241) was found. The intensity of CD248 was negatively correlated with age to a moderate degree (R = -0.407). Additionally, age was positively correlated with A lesion (R = 0.446).

Apart from age, A lesion and CNV area, two representative indicators of the severity of atrophic and neovascular lesions in our study were included in the correlation tests (Fig. 8). In terms of atrophic lesions, a positive correlation between A lesion and GFAP (R = 0.379) and EGFR (R = 0.291) was shown, and a negative correlation between A lesion and SFRP3 (R = -0.502), CD248 (R = -0.495) and COL6A2 (R = -0.309) was demonstrated. However, there was no significant correlation between CNV area and potential AH biomarkers (P > 0.05).

Additionally, a correlation was shown among potential AH biomarkers, including GFAP, EGFR, SFRP3, CD248, COL2A1, and COL6A2, with Pearson R ranging from − 0.55 to -0.241 and from 0.221 to 0.454 (Fig. 8).

## Discussion

In this cross-sectional study, we reported a proteomic analysis of patients with pathological myopia at different stages. Based on the new ATN classification system, myopic maculopathy (MM) involved 3 important pathological morphological characters, including myopic atrophy maculopathy (MAM), myopic tractional maculopathy (MTM) and myopic neovascular maculopathy (MNM). For preliminary research, we analyzed the demographic statistics of 188 high myopic eyes of 117 participants to specify the groups needed for subsequent proteomic experiments. A positive correlation was found between atrophic and neovascular lesions based on the ATN grading system for MM, but no correlation was observed between atrophic and tractional components or between neovascular and tractional components (Table 1). The proportion of MNM was larger with enlarged A lesion (Fig. 1). Our data were similar to those of previous studies. In a previous study, MAM and MNM were considered more relevant and had similar risk factors, whereas MTM presented distinctive risk factors and appeared to be more independent [26]. Other studies also supported that pmCNV, which was a type of MNM lesion (N2a stage) [24], was more likely to derive from atrophic lesions [27, 28].

Accordingly, we enrolled 3 groups, including the non-MM, MAM, and pmCNV groups, for proteomic testing. Several studies have investigated alterations in AH protein expression in human samples in myopic populations through high-throughput techniques [14, 15], but few have strictly classified the different types of MM depending on the practical and newest category—the ATN grading system [12, 24].

Subsequently, we found different patterns of protein changes in the comparison of pmCNV with other groups (Fig. 2). The DEP analyses suggested distinctive patterns of abundance of proteins in pmCNV group. In addition, the complement-associated molecules (Fig. 6) and the recursive intensity change of GFAP (Fig. 7) in AH samples indicated possible distinctions between G2 and G3 group. These discoveries are supported by the consistency of other studies demonstrating the association of MAM and complement pathways [29].

Thus, from the macroscopic scale of clinical demographic anatomizing to the microscopic scale of the AH protein profile, we hypothesized that pmCNV and MAM shared similar characterization in some way.

The potential biomarkers of pmCNV were critical for determining the pathological process, disease progression, and prognosis of pmCNV. The origin of the proteins or whether it is secreted might imply a different course of pathological changes. The origin of these DEPs was of vital importance since their transport implied the pathological mechanisms. Proteins could be produced by tissues within the eye and others entered from the plasma especially when blood-aqueous barrier broke down [30]. Considering the pathology of pmCNV, posterior segment was the region that should be more concerned. However, there are few studies exploring the source of retinal/choroidal protein entry in AH [31, 32]. Tabibian et al. [33] presented a hypothesis about potential flux between the AH and posterior segments. Weigers ligament might be compromised when rhegmatogenous retinal detachment occurred, thus promoting the flux. A similar mechanism might exist in pmCNV, since vitreous-retinal degeneration also appeared in such pathological process. Therefore, GFAP and other proteins, such as FTH1, EGFR, and DBH, that commonly exist in the retina or choroid could be explained [34–36].

After a comprehensive search of the literature and preliminary analyses, such as enrichment analysis (Fig. 4; Table 3) and correlation tests (Fig. 8), we identified several potential biomarkers of pmCNV. GFAP served as a signature of astrocytes and was expressed on astrocytes and Müller cells in the retina. In the central nervous system, astrocytes construct a blood-brain barrier and have a critical impact on axonal metabolic homeostasis [37]. After laser injury, hypercitrullinated GFAP polymers were found to accumulate in Mülller cell endfeet [38]. Hypercitrullination altered retinal immunogen, as anticitrullinated peptide antibodies would emerge then. Namely, the increase of Müller glia, characterized by the increase of GFAP, exerted a compensatory protective effect against retinal stress. It is worth noting that the role of GFAP has been elucidated in other fundus diseases. In detail, GFAP was assessed in retinal detachment, proliferative vitreoretinopathy, branch retinal venous occlusion, and even malignant melanoma, which illustrated a compromise of the blood–retinal barrier (BRB) [39–42]. Specifically, astrocytes could prompt angiogenesis and elicit inflammation through reactive astrocytosis [43–46]. During neuroinflammation including AMD, microglia to astrocyte signaling might modulate the crosstalk between the astrocyte and vascular endothelial cells, leading to the remodeling of neurovascular unit [47]. Hence, the activation of astrocytes is an important indicator of the process of retinal/choroidal neovascularization disease. Notably, upregulated GFAP were also investigated during the pathological changes of uveitis, a typical inflammatory course in the eye [48]. Indeed, GFAP could serve as an important blood biomarker for intracerebral hemorrhage [49]. Other brain and spinal disorders were also characterized by the alteration of GFAP in the serum or cerebrospinal fluid [37]. In brief, the presence of other pathologies involving inflammation or neurovascular uncoupling possibly confounded the utility of GFAP as a biomarker. Thereafter, we highlighted the importance of GFAP changes in the aqueous humor during MAM and MNM, meanwhile further investigations are indispensable.

Except for growth factors released from endothelial cells (ECs), adjacent cells affect the growth of vessels. The autocrine or paracrine activity of heparin-binding epidermal growth factor–like growth factor (HB-EGF) promoted the development of CNV by binding to the EGF receptor (EGFR) [50]. The EGFR/Gab1 pathway was suggested to be a critical component of retinal NV [51]. In the present study, the augmented expression of EGFR in MNM could be caused by the vascular abnormalities. SFRP3 and PPP2R1A were enriched in the WNT pathway, which accompanied the progression of myopia. While studies on retinal diseases seldomly touched PPPR1A, Zhu et al. [52] revealed that Rlbp1-DTA-TCF/LEF heterozygous mice with disrupted Mülller cells had decreased WNT pathway inhibitor SFRP3. From this prospective, PPPR1A might be considered a vital laboratory index similar to GFAP.

The role of SLIT2 has been clarified as a candidate in angiogenesis regulation. As such, SLIT2 N-terminal(SLIT2-N) could exert an inhibitory effect on CNV by activating the Robo4/Akt pathway, thus abolishing VEGF-induced cell proliferation [53]. Remarkably, SLIT2 overexpression tended to be an angiogenic factor in the CNV model by binding to Robo1 or Robo2 [54, 55]. The cell cycle of RPE cells could be changed by recombinant SLIT2-N protein, and this bioactive fragment modulated VEGF transcription and expression levels in RPE cells in a dose-dependent manner. Moreover, assisted by endophilin-A2, SLIT2 could change the phosphorylation site on VEGFR2, thereby untethering cells into a migratory state rather than proliferating. Intriguingly, the downregulation of SLIT2 here suggests that its complex activity through different receptors needs to be determined by further evidence in pmCNV.

With respect to the microenvironment around ECs, pericytes should not be neglected. One pioneering study of CD248, which is expressed in pericytes, suggested that CD248-/- mice exhibited deficiencies in retinal vessel regression when exposed to hypoxic conditions during postnatal development. Consistently, CD248 was significantly decreased in our analysis.

The homeostasis of dopamine (DA) and its receptors might play a crucial role in axial length elongation and consequently influence the advancement of myopia [56]. As the rate-limiting enzyme for the synthesis of DA, DBH possibly maintained the delicate balance of DA metabolism. Here, we found that the DBH was significantly increased in aqueous humor of G1 group. Other identified proteins of interest included two collagen proteins, COL2A1 and COL6A2. The sclera is mainly composed of collagen fibers and fibroblasts, and the hypoxic sclera is relevant to the pathogenesis of myopia [57, 58]. In addition, hypoxia exposure induced trans-differentiation into myofibroblasts and reduced the expression of COL1A1 in human scleral fibroblast cells [59]. The occurrence of our collagen proteins implied that pmCNV and pathologic sclera were interrelated, possibly highlighting a deeper and more complete breakdown of barriers in the eye.

Generally, VEGF and other angiogenesis factors had been highlighted in myopic neovascular research. Several studies had focused on the protein levels of VEGF and PEDF in the AH, but variations could be found in their conclusions (Supplementary Table S3). PEDF was upregulated in the AH of pmCNV eyes compared to that of non-MM eyes, but VEGF showed no significant difference between pmCNV and other groups here, thus making these factors limited for biomarkers due to their discrepancies.

Altogether, these findings tend to verify our hypothesis on the similarities of MAM and pmCNV by the comparability of molecule alterations during myopia. Despite the limited number of patients enrolled, we recommend potential biomarkers for pmCNV. More mechanistic studies are imperative and should concentrate on damage to the BRB and other vascular growth factors in addition to VEGF.

## Methods

### Subjects

Ethical approval was granted by the medical ethics committee of Tongji Hospital, Tongji Medical College, Huazhong University of Science and Technology. The study adhered to the Declaration of Helsinki guidelines and was registered at http://www.chictr.org.cn (registration number ChiCTR2100043611, ChiCTR2100046511). All participants gave written informed consent.

A total of 188 highly myopic eyes (117 participants) were enrolled, the inclusion and exclusion criteria were same as previously described [25].The patients received history review and their ocular examinations were collected. Among these patients, 24 eyes underwent cataract surgery during the study period, and 19 treatment-naïve mCNV eyes received anti-VEGF therapy. Before and after the surgery, best-corrected visual acuity (BCVA), color fundus photography (AFC-210, Nidek Co., LTD, Japan), optical coherence tomography (OCT), and OCT angiography (Spectralis OCT, Heidelberg Engineering, Germany and SVision Imaging, Henan, China) examinations were conducted.

### Classification of myopic maculopathy and groups

According to the ATN classification system [24], two specialists diagnosed the fundus examination images and graded myopic maculopathy, and disagreements were adjudicated by a more experienced retinal professor. In detail, a degree greater than 1 was defined as MAM, which included diffuse atrophy (A2), patchy atrophy (A3), and macular atrophy (A4); A0 had no atrophic lesions, and A1 was a tessellated lesion. T components were T0 (no macular schisis), T1 (inner or outer foveoschisis), T2, inner and outer foveoschisis, T3 (foveal retinal detachment), T4 (full-thickness macular hole [MH]), and T5 (MH and retinal detachment). A degree equal to or greater than 1 was classified as MNM, which was composed of a lacquer crack (N1), active pmCNV (N2a), scar or Fuch’s spot (N2s).

Thereafter, 24 eyes were selected for further analysis and divided into different groups: the G1 group contained 12 treatment-naïve pmCNV eyes (N2a), the G2 group contained 6 eyes with myopic atrophic maculopathy (MAM, A ≥ 2), whereas the G3 group contained 6 eyes without myopic maculopathy (non-MM, A0T0N0 or A1T0N0).

### AH collection

AH samples of the control group were collected from eyes with cataracts before surgery; AH samples of the experimental group were collected from treatment-naïve mCNV eyes before the first intravitreal injection of conbercept. Samples (70–150 µl) were collected in Eppendorf tubes and stored at -80 °C until further analysis.

### Proteomics

The cellular debris from AH sample was removed by centrifugation at 12,000 g at 4 °C for 10 min. Then, the supernatant was transferred to a new centrifuge tube. The most abundant 14 proteins in human serum (human serum albumin, albumin, IgG, IgA, IgM, IgD, kappa and lambda light chains of IgE, alpha-1-acidglycoprotein, alpha-1-antitrypsin, alpha-2-macroglobulin, apolipoprotein A1, fibrinogen, haptoglobin, and transferrin) were removed by Pierce™ Top 14 Abundant Protein Depletion Spin Columns Kit (ThermoFisher Scientific, Waltham, MA, USA). Then, the protein concentration was quantified by a BCA kit (ThermoFisher Scientific). Before digestion, the protein solution was reduced with 5 mM dithiothreitol for 30 min at 56 °C and alkylated with 11 mM iodoacetamide for 15 min at room temperature in darkness. The protein sample was then diluted by adding 100 mM Tetraethylammonium Bromide (TEAB) until urea concentration was less than 2 M. Trypsin was added (1:50 trypsin-to-protein mass ratio) for a first digestion overnight (14-16 h) -digestion and then (1:100 trypsin-to-protein mass ratio) for a subsequent 4 h-digestion.

After digestion into peptides, the samples were dissolved in solvent A (0.1% formic acid, 2% acetonitrile/in water) and loaded onto a home-made reversed-phase analytical column (25-cm length, 75 μm i.d.). Peptides were separated with a gradient from 5 to 25% solvent B (0.1% formic acid in 90% acetonitrile) over 90 min, 25–35% in 22 min and ascending 80% in 4 min then holding at 80% for the last 4 min, all at a constant flowrate of 450 nL/min on an EASY-nLC 1200 UPLC system (Thermo Fisher Scientific).

The separated peptides were analyzed in Exploris 480TM (Thermo Fisher Scientific) with a nano-electrospray ion source. The electrospray voltage applied was set as 2.2 kV and the compensation voltages was − 35 V. The full MS scan resolution was set to 60,000 for a scan range of 400–1200 m/z. The precursors underwent 20s dynamic exclusion, and selected for next higher energy collision induced fragmentation (HCD) procedure at a normalized collision energy (NCE) of 35%. The fragments were detected in the Orbitrap at a resolution of 15,000.

### Proteome database search

The resulting MS/MS data were processed using MaxQuant search engine (v.1.6.15.0). Tandem mass spectra were searched against the human SwissProt database (20,422 entries) concatenated with reverse decoy database. Trypsin (Full) was specified as cleavage enzyme allowing up to 2 missing cleavages. The mass tolerance for precursor ions was set as 20 ppm in first search and 5 ppm in main search, and the mass tolerance for fragment ions was set as 0.02 Da. Carbamidomethyl on Cys was specified as fixed modification, and acetylation on protein N-terminal and oxidation on Met were specified as variable modifications. To ensure a higher-quality result, FDR was adjusted to < 1%.

### Bioinformatic analysis

Gene Ontology (GO) annotation proteome was derived from the UniProt-GOA database (http://www.ebi.ac.uk/GOA/). Identified protein ID was matched to Uniprot ID and then mapping to GO IDs by protein ID through application of eggnog-mapper (v2.0). The unannotated proteins in Uniprot-GOA database would be explored by the InterProScan soft based on protein sequence alignment method. Proteins were classified by GO annotation into three categories: biological process, cellular compartment and molecular function. For each category, a two-tailed Fisher’s exact test was employed to test the enrichment of the differentially expressed protein against all identified proteins. The GO with a corrected p-value < 0.05 is considered significant.

### Statistical analysis

In the highly myopic cohort, the distribution of different MM types is shown in Fig. 1, and correlations among the 3 types of MM were analyzed using Spearman correlation analysis (Table 1). Comparison of clinical characteristics among 24 patients whose samples were used for proteomics analysis was performed (Table 2). Statistical analyses in the study were performed with R (http://www.R-project.org) and Empower Stats software (www.empowerstats.com. X&Y solutions, Inc. Boston MA), or GraphPad Prism 8 software.

To determine the protein expression difference, a fold change > 1.5 and P < 0.05 were set in comparison of the average between two groups. Overlapping DEPs in two comparisons (G1 vs. G2, G1 vs. G3) were selected for further analysis (Figs. 2 and 3). Enrichment analysis was performed based on Gene Ontology (GO) and Kyoto Encyclopedia of Genes and Genomes (KEGG) annotation; a P < 0.05 was regarded as the significance standard in Fisher’s exact test (Table 3). The relative intensity of DEPs among the 3 groups was demonstrated in graphs using GraphPad Prism 8 software (Figs. 6 and 7).

### Electronic supplementary material

Below is the link to the electronic supplementary material.

Supplementary Material 1

Supplementary Material 2

Supplementary Material 3

Supplementary Material 4

Supplementary Material 5

Supplementary Material 6

Supplementary Material 7

## Data Availability

All mass spectrometry proteomics raw data have been deposited to the PRIDE database with data set identifier PXD038430.

## Abbreviations

pmCNV: Pathologic myopia choroidal neovascularization
AH: Aqueous humor
MM: Myopic maculopathy
MAM: Myopic atrophic maculopathy
MTM: Myopic tractional maculopathy
MNM: Myopic neovascular maculopathy
PM: Pathologic myopia
CNV: Choroidal neovascularization
VEGF: Vascular endothelial growth factor
AMD: Aged-related macular degeneration
BCVA: Best-corrected visual acuity
OCT: Optical coherence tomography
non-MM: Without myopic maculopathy
GO: Gene ontology
KEGG: Kyoto Encyclopedia of Genes and Genomes
DEPs: Differentially expressed proteins
