# Supplementary material for: Insights into myopic choroidal neovascularization based on quantitative proteomics analysis of the aqueous humor
Source: BMC Genomics. 2023 Dec 12;24:767. doi: 10.1186/s12864-023-09761-z (PMC10714574; doi:10.1186/s12864-023-09761-z)

**Supplementary Figure S1** Differentially expressed proteins in AH between MAM group and non-MM group.


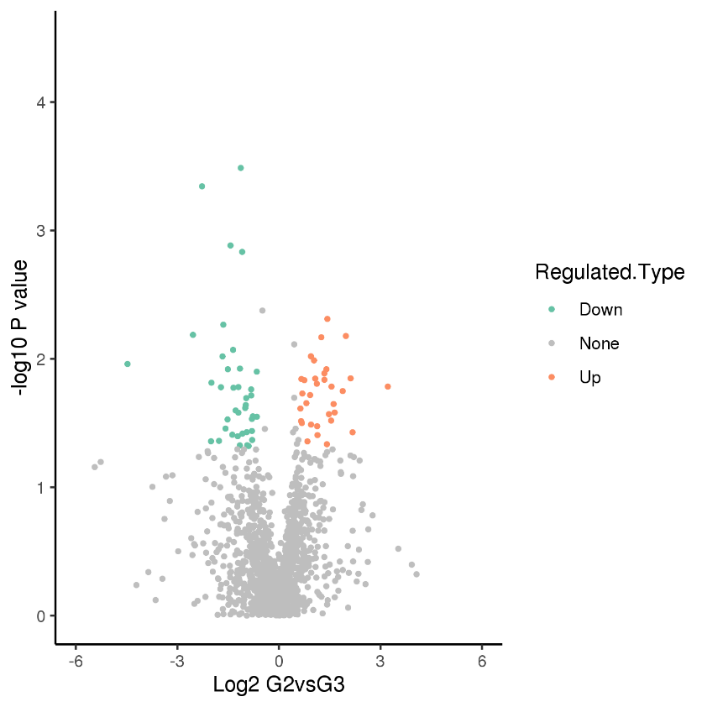

Supplement: Supplementary file 1 — Supplementary Material 1 [file 12864_2023_9761_MOESM1_ESM.docx]
