# Supplementary material for: Insights into myopic choroidal neovascularization based on quantitative proteomics analysis of the aqueous humor
Source: BMC Genomics. 2023 Dec 12;24:767. doi: 10.1186/s12864-023-09761-z (PMC10714574; doi:10.1186/s12864-023-09761-z)
Supplement: Supplementary file 2 — Supplementary Material 2 [file 12864_2023_9761_MOESM2_ESM.docx]

**Supplementary Table S1** Upregulated proteins in AH of MAM group compared to non-MM group.

| Protein Name | Gene Name | G2/G3 ratio | P value | Trend |
| --- | --- | --- | --- | --- |
| Protein MGARP | MGARP | 9.295 | 0.016445109 | Up |
| Serpin B5 | SERPINB5 | 4.515 | 0.037351214 | Up |
| Tenascin-X | TNXB | 4.337 | 0.01418188 | Up |
| Glial fibrillary acidic protein | GFAP | 3.936 | 0.006641508 | Up |
| Vinculin | VCL | 3.689 | 0.017832581 | Up |
| 5'-3' exonuclease PLD4 | PLD4 | 3.127 | 0.026153887 | Up |
| Fatty acid-binding protein, liver | FABP1 | 3.062 | 0.022471504 | Up |
| Mesothelin (Fragment) | MSLN | 2.935 | 0.016461233 | Up |
| Alpha-(1,3)-fucosyltransferase 11 | FUT11 | 2.911 | 0.030269387 | Up |
| Matrix-remodeling-associated protein 5 | MXRA5 | 2.781 | 0.027003949 | Up |
| Agrin OS=Homo sapiens | AGRN | 2.695 | 0.00488649 | Up |
| Selenoprotein W | SELENOW | 2.67 | 0.046230977 | Up |
| Endoplasmic reticulum resident protein 29 | ERP29 | 2.643 | 0.012062076 | Up |
| Alpha-parvin (Fragment) | PARVA | 2.535 | 0.014540601 | Up |
| T-complex protein 1 subunit beta | CCT2 | 2.533 | 0.01296863 | Up |
| DNA dC->dU-editing enzyme APOBEC-3B | APOBEC3B | 2.381 | 0.006785201 | Up |
| Fibrinogen alpha chain | FGA | 2.2 | 0.039220156 | Up |
| N-acetyllactosaminide beta-1,3-N-acetylglucosaminyltransferase 2 | B3GNT2 | 2.187 | 0.03352999 | Up |
| Kallikrein-11 | KLK11 | 2.172 | 0.015630379 | Up |
| HLA class I histocompatibility antigen, C alpha chain | HLA-C | 2.103 | 0.014247442 | Up |
| Complement C1q subcomponent subunit A | C1QA | 2.051 | 0.0102788 | Up |
| Complement component C8 alpha chain | C8A | 1.929 | 0.032488218 | Up |
| C1QTNF3-AMACR readthrough (NMD candidate) | C1QTNF3-AMACR | 1.925 | 0.009554885 | Up |
| Complement C1q subcomponent subunit C | C1QC | 1.894 | 0.019162034 | Up |
| Zinc finger protein 185 | ZNF185 | 1.791 | 0.043954823 | Up |
| Cathepsin O | CTSO | 1.753 | 0.022135397 | Up |
| Mannosyl-oligosaccharide 1,2-alpha-mannosidase IB | MAN1A2 | 1.682 | 0.01466313 | Up |
| Protein eyes shut homolog | EYS | 1.614 | 0.018642315 | Up |
| Reelin | RELN | 1.599 | 0.031638212 | Up |
| Alpha-1-antichymotrypsin | SERPINA3 | 1.583 | 0.014355224 | Up |
| Cathepsin F | CTSF | 1.574 | 0.030567446 | Up |
| Complement component C7 | C7 | 1.552 | 0.02439317 | Up |
