# Supplementary material for: Insights into myopic choroidal neovascularization based on quantitative proteomics analysis of the aqueous humor
Source: BMC Genomics. 2023 Dec 12;24:767. doi: 10.1186/s12864-023-09761-z (PMC10714574; doi:10.1186/s12864-023-09761-z)
Supplement: Supplementary file 3 — Supplementary Material 3 [file 12864_2023_9761_MOESM3_ESM.docx]

**Supplementary Table S2** Downregulated proteins in AH of MAM group compared to non-MM group.

| Protein Name | Gene Name | G2/G3 ratio | P value | Trend |
| --- | --- | --- | --- | --- |
| Myotrophin | MTPN | 0.045 | 0.010965152 | Down |
| Immunoglobulin lambda variable 4-69 | IGLV4-69 | 0.172 | 0.006501533 | Down |
| Metallothionein-1X | MT1X | 0.207 | 0.000453308 | Down |
| Keratinocyte proline-rich protein | KPRP | 0.249 | 0.04380293 | Down |
| Keratin, type I cytoskeletal 15 | KRT15 | 0.251 | 0.015363186 | Down |
| Glutathione reductase, mitochondrial | GSR | 0.293 | 0.043565643 | Down |
| 40S ribosomal protein S30 | FAU | 0.305 | 0.01665105 | Down |
| ZNF511-PRAP1 readthrough (Fragment) | ZNF511-PRAP1 | 0.315 | 0.009563868 | Down |
| Proteasome subunit alpha type (Fragment) | PSMA4 | 0.32 | 0.005424498 | Down |
| Galactose mutarotase | GALM | 0.335 | 0.034904359 | Down |
| Immunoglobulin kappa joining 1 | IGKJ1 | 0.349 | 0.029657851 | Down |
| Malate dehydrogenase | MDH1 | 0.351 | 0.012060859 | Down |
| Extracellular sulfatase Sulf-1 | SULF1 | 0.371 | 0.001309733 | Down |
| WAP four-disulfide core domain protein 1 | WFDC1 | 0.385 | 0.038968321 | Down |
| Cocaine- and amphetamine-regulated transcript protein | CARTPT | 0.391 | 0.008504733 | Down |
| Glyoxalase domain-containing protein 4 | GLOD4 | 0.394 | 0.016776953 | Down |
| All-trans-retinol dehydrogenase [NAD(+)] ADH7 | ADH7 | 0.412 | 0.025231161 | Down |
| N(G),N(G)-dimethylarginine dimethylaminohydrolase 1 | DDAH1 | 0.43 | 0.040086353 | Down |
| Dipeptidyl peptidase 3 (Fragment) | DPP3 | 0.436 | 0.016594006 | Down |
| Titin | TTN | 0.436 | 0.026267122 | Down |
| Membrane-associated progesterone receptor component 1 | PGRMC1 | 0.449 | 0.047169141 | Down |
| Multiple epidermal growth factor-like domains protein 11 | MEGF11 | 0.45 | 0.011919789 | Down |
| Aspartate aminotransferase, cytoplasmic | GOT1 | 0.458 | 0.000325936 | Down |
| 5'-3' exonuclease PLD3 | PLD3 | 0.471 | 0.001467435 | Down |
| Aldo-keto reductase family 1 member A1 | AKR1A1 | 0.474 | 0.038160004 | Down |
| Iduronate 2-sulfatase | IDS | 0.501 | 0.024176823 | Down |
| Melanoma antigen recognized by T-cells 1 | MLANA | 0.507 | 0.022888923 | Down |
| Contactin-4 | CNTN4 | 0.512 | 0.020244132 | Down |
| Acyl-CoA-binding protein | DBI | 0.517 | 0.037278827 | Down |
| Gamma-crystallin C | CRYGC | 0.522 | 0.047126955 | Down |
| Superoxide dismutase [Cu-Zn] | SOD1 | 0.537 | 0.047742884 | Down |
| Coiled-coil domain-containing protein 187 | CCDC187 | 0.566 | 0.017304394 | Down |
| EGF-containing fibulin-like extracellular matrix protein 1 (Fragment) | EFEMP1 | 0.567 | 0.019264235 | Down |
| Sialomucin core protein 24 | CD164 | 0.575 | 0.029371698 | Down |
| V-set and transmembrane domain-containing protein 2-like protein | VSTM2L | 0.575 | 0.036446923 | Down |
| Xanthine dehydrogenase/oxidase | XDH | 0.578 | 0.042827242 | Down |
| Protein sel-1 homolog 1 | SEL1L | 0.586 | 0.02807459 | Down |
| Alpha-1B-glycoprotein (Fragment) | A1BG | 0.635 | 0.012583263 | Down |
| Ras-related protein Rab-14 (Fragment) | RAB14 | 0.636 | 0.028263941 | Down |
