# Supplementary material for: Insights into myopic choroidal neovascularization based on quantitative proteomics analysis of the aqueous humor
Source: BMC Genomics. 2023 Dec 12;24:767. doi: 10.1186/s12864-023-09761-z (PMC10714574; doi:10.1186/s12864-023-09761-z)
Supplement: Supplementary file 4 — Supplementary Material 4 [file 12864_2023_9761_MOESM4_ESM.docx]

**Supplementary Table 5** Clinical characteristics and association with biomarkers

| Patient | Central macular thickness(μm) | Macular choroidal thickness(μm) | CNV volume(mm2) | FCN3 | GFAP | EGFR | SFRP3 | PPP2R1A | SLIT2 | CD248 | DBH | COL2A1 | COL6A2 |
| --- | --- | --- | --- | --- | --- | --- | --- | --- | --- | --- | --- | --- | --- |
| 1-1 | 103 | 14 | 0.39 | 1.51 | 2.564 | 2.215 | 0.596 | 0.817 | 0.575 | 0.66 | 1.666 | 0.505 | 0.02 |
| 1-2 | 40 | 14 | 0.71 | 2.007 | 3.946 | 0.94 | 0.288 | 0.556 | 0.131 | 0.495 | 2.077 | 0.239 | 0.035 |
| 1-3 | 338 | 19 | 1.33 | 2.786 | 0.932 | 1.737 | 0.787 | 0.943 | 0.329 | 1.034 | - | 0.729 | 1.084 |
| 1-4 | 219 | 126 | 0.32 | 0.946 | 3.624 | 2.17 | 0.227 | 0.966 | - | 1.189 | - | 0.253 | 0.008 |
| 1-5 | 500 | 52 | 1.88 | 1.204 | 0.854 | 1.226 | 1.466 | - | 1.311 | 0.7 | - | 1.056 | 0.577 |
| 1-6 | 188 | 43 | 0.5 | 2.566 | 1.671 | 2.536 | 0.638 | - | 0.3 | 0.713 | - | 0.599 | 0.177 |
| 1-7 | 219 | 135 | 0.018 | 1.554 | 3.487 | 0.83 | 1.819 | - | 0.663 | 0.327 | - | 1.884 | 0.487 |
| 1-8 | 231 | 50 | 0.015 | 1.298 | 1.351 | 0.937 | 0.95 | - | 0.297 | 0.777 | - | 0.62 | 0.412 |
| 1-9 | - | 111 | - | 1.328 | 4.944 | 2.45 | 0.458 | - | 0.294 | 1.052 | - | 0.616 | 0.408 |
| 1-10 | 97 | 17 | 3.24 | 1.423 | 0.527 | 1.964 | 0.975 | 0.576 | - | 0.988 | 2.62 | 1.507 | 0.232 |
| 1-11 | 184 | 35 | 1.15 | 1.825 | 2.683 | 1.99 | 0.358 | 1.106 | - | 0.389 | 2.964 | 0.721 | 0.116 |
| 1-12 | 95 | 2 | - | 1.318 | 1.913 | 1.991 | 0.392 | 0.892 | 1.517 | 0.708 | - | 0.329 | 0.129 |
| 2-1 |  |  |  | 0.981 | 1.71 | 0.488 | 0.799 | 1.411 | 1.471 | 0.761 | - | 0.858 | 4.896 |
| 2-2 |  |  |  | 1.004 | 0.543 | 1.028 | 1.303 | 1.11 | 1.933 | 0.975 | - | 2.199 | 0.199 |
| 2-3 |  |  |  | 0.566 | 1.179 | 1.785 | 1.336 | 1.758 | 0.681 | 0.973 | - | 1.178 | 0.637 |
| 2-4 |  |  |  | 1.968 | 1.111 | 0.803 | 1.072 | 0.991 | 2.494 | 1.742 | - | 1.001 | 1.25 |
| 2-5 |  |  |  | - | 0.25 | 0.311 | 1.416 | 1.59 | 0.5 | 2.513 | 0.993 | 2.484 | 1.297 |
| 2-6 |  |  |  | 0.476 | 1.677 | 0.897 | 1.478 | 0.919 | 0.785 | 1.232 | 1.116 | 1.275 | 1.112 |
| 3-1 |  |  |  | - | 0.138 | 1.113 | 1.668 | 1.629 | 0.72 | 3.434 | 0.211 | 1.803 | 0.689 |
| 3-2 |  |  |  | 0.902 | 0.083 | 0.626 | 1.727 | - | 1.563 | 1.962 | - | 1.133 | 0.808 |
| 3-3 |  |  |  | 1.607 | 0.12 | 1.502 | 0.978 | 0.934 | - | 1.346 | - | 1.144 | 0.132 |
| 3-4 |  |  |  | - | 1.203 | 1.325 | 1.593 | 2.864 | 1.044 | 1.089 | 0.63 | 1.934 | 1.075 |
| 3-5 |  |  |  | 0.853 | 0.057 | 0.353 | 4.796 | - | 1.966 | 0.747 | - | 1.245 | 6.166 |
| 3-6 |  |  |  | 0.527 | 0.043 | 0.851 | 0.583 | - | 2.985 | 1.051 | 0.66 | 1.84 | 4.909 |
