# Supplementary material for: Insights into myopic choroidal neovascularization based on quantitative proteomics analysis of the aqueous humor
Source: BMC Genomics. 2023 Dec 12;24:767. doi: 10.1186/s12864-023-09761-z (PMC10714574; doi:10.1186/s12864-023-09761-z)
Supplement: Supplementary file 5 — Supplementary Material 5 [file 12864_2023_9761_MOESM5_ESM.docx]

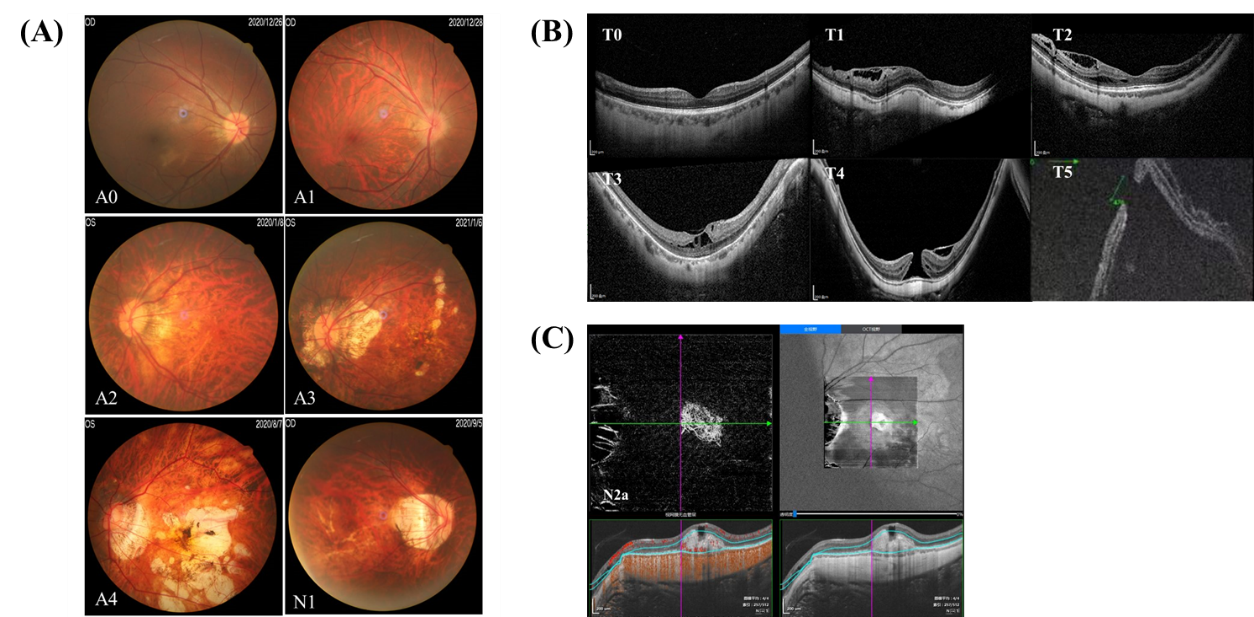


**Figure S2.** Fundus images of different types of lesion in ATN classification system.

(A) Different types of A lesions and N lesions. (A0) No myopic retinal lesions. (A1) Tesselated fundus only. (A2) Diffuse chorioretinal atrophy. (A3) Patchy chorioretinal atrophy. (A4) Complete macular atrophy. Also, Fuch’s spot (dark lesion in atrophy area) was also shown (N2s). (N1) Macular lacquer cracks.

(B) Different types of T lesions. (T0) No macular schisis. (T1) Inner foveoschisis. (T2) Inner and outer foveoschisis. (T3) Foveal detachment. (T4) Full-thickness MH.

(C) OCTA image of an active CNV (N2a lesion) patient. In the avascular layer, OCTA image showed a neovascular (top left). And the B-scan image showed blood signal (red points in bottom left) in the neovascular.
