# Supplementary material for: Insights into myopic choroidal neovascularization based on quantitative proteomics analysis of the aqueous humor
Source: BMC Genomics. 2023 Dec 12;24:767. doi: 10.1186/s12864-023-09761-z (PMC10714574; doi:10.1186/s12864-023-09761-z)
Supplement: Supplementary file 6 — Supplementary Material 6 [file 12864_2023_9761_MOESM6_ESM.docx]

**Supplementary Table S3** Potential biomarkers explored in previous studies.

| Article | Source | Method | Experimental group | Control group | DEPs | trend |
| --- | --- | --- | --- | --- | --- | --- |
| [12] | AH | ELISA | pmCNV (21 treatment naïve eyes) | senile cataract (21 eyes) | VEGF | downregulated |
| [13] | AH | ELISA | pmCNV (9 treatment naïve eyes) | senile cataract (10 eyes) | VEGF，PEDF | upregulated |
| [14] | AH | ELISA | pmCNV (20 treatment naïve eyes) | senile cataract (20 eyes) | VEGF，PEDF | downregulated |
| [15] | AH | Luminex 200 | pmCNV (30 treatment naïve eyes) | senile cataract (43 eyes) | VEGF | No difference |
|  |  |  |  |  | IL-8，IL-10， MCP-1 | upregulated |
| [16] | AH | Bio-Plex ^TM^ Human Cytokine 27-Plex panel | pmCNV (19 treatment naïve eyes) | Highly myopic eyes (15) | VEGF | downregulated |
|  |  |  |  |  | PDGF，IL-2，IL-5，IL-13， IL-15, IL-17A， TNF-α，IL-8，RANTES | upregulated |
| [17] | Serum | Immne nephelometry | pmCNV patients (24) | emmetropic people (51) | C3 | upregulated |
| [18] | VH | Proteomics | pmCNV and PMRS, after IVC treatment (3 eyes) | PMRS without pmCNV (3 eyes) | α-SMA | upregulated |
|  |  |  |  |  |  | downregulated |
|  |  |  | pmCNV and PMRS (4 eyes) | PMRS without pmCNV (3 eyes) | aA-crystallin, fibrillin-1 | upregulated |
