# Supplementary material for: Insights into myopic choroidal neovascularization based on quantitative proteomics analysis of the aqueous humor
Source: BMC Genomics. 2023 Dec 12;24:767. doi: 10.1186/s12864-023-09761-z (PMC10714574; doi:10.1186/s12864-023-09761-z)
Supplement: Supplementary file 7 — Supplementary Material 7 [file 12864_2023_9761_MOESM7_ESM.docx]

**Supplementary Table S4** clinical data and ATN degree for the enrolled patients

| Group | Patient | Gender | Eye | Age | Axial length | Atrophic component | Tractional component | Neovascular component | Chief compliant and duration for pmCNV |
| --- | --- | --- | --- | --- | --- | --- | --- | --- | --- |
| G1-pmCNV | 1-1 | female | OD | 58 | 32.67 | 3 | 0 | 2a | blurred vision (1mon) |
| G1-pmCNV | 1-2 | female | OD | 64 | 30.32 | 4 | 0 | 2a | decreased visual acuity (1mon) |
| G1-pmCNV | 1-3 | female | OD | 69 | 28.06 | 2 | 2 | 2a | shadow(1mon) |
| G1-pmCNV | 1-4 | male | OS | 56 | 28.00 | 3 | 1 | 2a | decreased visual acuity(1mon) |
| G1-pmCNV | 1-5 | female | OS | 55 | 27.11 | 2 | 1 | 2a | blurred vision(1w) |
| G1-pmCNV | 1-6 | female | OD | 66 | 29.20 | 2 | 1 | 2a | NA |
| G1-pmCNV | 1-7 | female | OD | 51 | 28.02 | 1 | 0 | 2a | blurred vision(0.5mon) |
| G1-pmCNV | 1-8 | female | OS | 67 | 28.21 | 4 | 2 | 2a | blurred vision(2d) |
| G1-pmCNV | 1-9 | male | OS | 46 | 32.24 | 2 | 0 | 2a | blurred vision(2w) |
| G1-pmCNV | 1-10 | male | OD | 75 | 29.38 | 4 | 0 | 2a | decreased visual acuity(1y) |
| G1-pmCNV | 1-11 | male | OS | 75 | 30.79 | 3 | 1 | 2a | decreased visual acuity(1y) |
| G1-pmCNV | 1-12 | female | OS | 52 | 31.38 | 4 | 1 | 2a | NA |
| G2-MAM | 2-1 | male | OS | 52 | 35.21 | 3 | 0 | 0 | - |
| G2-MAM | 2-2 | female | OD | 49 | 29.47 | 3 | 0 | 0 | - |
| G2-MAM | 2-3 | female | OS | 42 | 28.07 | 2 | 0 | 0 | - |
| G2-MAM | 2-4 | male | OS | 47 | 33.36 | 2 | 0 | 0 | - |
| G2-MAM | 2-5 | female | OD | 51 | 33.16 | 2 | 0 | 0 | - |
| G2-MAM | 2-6 | female | OS | 50 | 27.83 | 2 | 0 | 0 | - |
| G3-non-MM | 3-1 | female | OS | 38 | 27.89 | 0 | 0 | 0 | - |
| G3-non-MM | 3-2 | female | OD | 47 | 26.50 | 0 | 0 | 0 | - |
| G3-non-MM | 3-3 | male | OD | 48 | 26.16 | 1 | 0 | 0 | - |
| G3-non-MM | 3-4 | female | OS | 43 | 30.14 | 1 | 0 | 0 | - |
| G3-non-MM | 3-5 | male | OD | 52 | 30.95 | 1 | 0 | 0 | - |
| G3-non-MM | 3-6 | male | OD | 55 | 27.50 | 1 | 0 | 0 | - |
